# Understanding Who Benefits from Parenting Interventions for Children’s Conduct Problems: an Integrative Data Analysis

**DOI:** 10.1007/s11121-018-0864-y

**Published:** 2018-01-18

**Authors:** Patty Leijten, Maartje Raaijmakers, Leoniek Wijngaards, Walter Matthys, Ankie Menting, Maud Hemink-van Putten, Bram Orobio de Castro

**Affiliations:** 1 University of Amsterdam, Amsterdam, Netherlands; 2 Utrecht University, Utrecht, Netherlands

**Keywords:** Conduct problems, Parenting intervention, Diversity, Integrative data analysis

## Abstract

Parenting interventions are an effective strategy to reduce children’s conduct problems. For some families, that is, not all families benefit equally. Individual trials tend to be underpowered and often lack variability to differentiate between families how benefit less or more. Integrating individual family level data across trials, we aimed to provide more conclusive results about often presumed key family (parental education and ethnic background) and child characteristics (problem severity, ADHD symptoms and emotional problems) as putative moderators of parenting intervention effects. We included data from 786 families (452 intervention; 334 control) from all four trials on the Incredible Years parenting intervention in The Netherlands (three randomized; one matched control). Children ranged between 2 and 10 years (*M* = 5.79; *SD* = 1.66). Of the families, 31% had a lower educational level and 29% had an ethnic minority background. Using multilevel regression, we tested whether each of the putative moderators affected intervention effects. Incredible Years reduced children’s conduct problems (*d* = − .34). There were no differential effects by families’ educational or ethnic background, or by children’s level of ADHD symptoms. Children with more severe conduct problems and those with more emotional problems benefited more. Post hoc sensitivity analyses showed that for the two trials with longer-term data, moderation effects disappeared at 4 or 12 months follow-up. Often assumed moderators have some, but limited abilities to explain who benefits from parenting interventions. This suggests the need for studying theoretically more precise moderators in prevention research, other than relatively static family characteristics alone.

Parenting interventions can effectively prevent and reduce conduct problems in children (Weisz and Kazdin 2010). Yet, about a quarter to a third of the families fails to show improvement from established parenting interventions (Scott et al. 2001; Shelleby and Shaw 2014). Our ability to predict who will benefit more from prevention efforts, and who will benefit less, are limited (Ng and Weisz 2016). Most individual trials have insufficient statistical power and variance to predict who benefits (Brown et al. 2013). Yet, identifying the families who benefit is critical for understanding why conduct problems can be changed successfully by providing parenting support in some families and not in other families (Rutter and Pickles 2016). This understanding in turn is vital for strengthening prevention strategies to reduce children’s conduct problems.

In this study, we aimed to shed light on the extent to which widely studied moderators explain for whom parenting interventions reduce conduct problems, by synthesizing individual family level data across four trials to increase statistical power and variance. Many different putative moderators have been suggested (Shelleby and Shaw 2014). We included family and child characteristics as moderators that (1) are key predictors of conduct disorder, to examine whether particularly children who are most at risk benefit less or more from prevention efforts, and (2) suffer from inconsistent findings in individual trials and meta-analyses about their impact on intervention effectiveness.

## Parental Educational Level and Ethnic Background

Low parental educational level impacts child development in part through the chronic stressors that often accompany the lower socioeconomic status of parents with lower educational levels, such as deprived neighborhoods and parental mental health problems (e.g., Conger et al. 1992). Similar stressors are suggested to explain why lower educated families may benefit less from parenting interventions. Empirical findings, however, vary between showing that socioeconomically disadvantaged family benefit less (e.g., Leijten et al. 2012), equally (Gardner et al. 2010), or more (e.g., Gardner et al. 2009; MacKenzie et al. 2004). Perhaps even more surprising are the equally inconsistent findings from meta-analyses (e.g., Deković et al. 2011; Leijten et al. 2013; Lundahl et al. 2006). In sum, parental educational level as part of family socioeconomic status is studied exhaustively, but its role in parenting intervention effectiveness remains unclear.

Children from ethnic minority families with a relatively recent immigration history in Western Europe are at higher risk for the development of conduct disorder (e.g., Sagatun et al. 2008). Besides, culture and language differences may impact to what extent families benefit from interventions. One previous study integrated data from two parenting intervention trials (Reid et al. 2001) and found no meaningful differences in intervention benefits across ethnic groups in the USA. Individual trials in Europe (e.g., Bjørknes and Manger 2013) and a meta-analysis (Menting et al. 2013) reached similar conclusions. Despite these findings, concerns remain that ethnic minority populations, particularly those with relatively recent migration histories, may benefit less from parenting interventions that are developed in dominant cultural groups in North America and Western Europe (e.g., Miranda et al. 2005).

## Conduct Problem Severity and Associated Problems

Conduct problems in early childhood is the strongest predictor of conduct disorders in later life (Moffitt and Caspi 2001). Most individual trials and meta-analyses suggest that children with more severe conduct problems benefit more from parenting interventions (e.g., Leijten et al. 2013; Menting et al. 2013). Mechanisms underlying this effect may be a larger scope for improvement and increased parental motivation to change. In some trials, however, children with more severe conduct problems benefited less (e.g., Kazdin and Wassell 2000). Inconsistencies might be explained by limited variance in problem severity within individual trials; trials tend to focus on either prevention or treatment of conduct disorder and rarely include children with a wide range of conduct problems.

A profound concern for prevention is that mental health problems often co-occur. ADHD symptoms and emotional problems are relatively common in children with conduct problems and are related to worse prognosis (Hinshaw et al. 1993; Rutter et al. 2006). The what extent children’s associated problems affect intervention outcomes is largely unknown. Systematic reviews (e.g., Ollendick et al. 2008; Riosa et al. 2011) show that very few trials include co-occurrence of mental health problems as a moderator of intervention effects. Some trials include co-occurring mental health problems as a predictor of intervention effects, but predictor analyses fail to correct for change in the control condition and can lead to misleading results (e.g., see Halldorsdottir and Ollendick 2016, for a comparison of predictor and moderator analysis). Findings from the few available tests of co-occurring mental health problems as moderators of intervention effectiveness are mixed. Some suggest that ADHD symptoms do not influence effectiveness (e.g., Bjørnebekk et al. 2015), or that children with more associated emotional problems benefit more (Jarrett et al. 2014).

## Why Integrate Data from Multiple Trials?

Individual trials on the effectiveness of parenting interventions tend to be powered to test main effects of the intervention, rather than moderation effects. Moreover, they are conducted in a certain time period and geographical region, and families are enrolled through a specific set of recruitment methods (Brown et al. 2013). This lack of statistical power and limited variance might explain why moderator findings are so inconsistent across trials. Meta-analyses are usually even more severely underpowered than individual trials to test moderation effects. Meta-analyses assess moderators at a trial level (e.g., the mean educational level of a sample) rather than at an individual level (e.g., the educational level of a parent). This means that the sample size of a meta-analysis equals the number of included trials, often a few dozen, and that all within trial variance is ignored (Lipsey 2003).

An alternative to traditional meta-analysis is integrative data analysis, or individual participant data meta-analysis (Curran and Hussong 2009). This approach synthesizes individual family level data from multiple trials and therefore uses variance between and within trials, gaining valuable power and generalizability. This highly recommended approach is rarely used in the social sciences because it requires authors to share their raw data, and because synthesizing data across trials can be difficult if different measures are used.

## The Present Study

We synthesized data from all trials on the effectiveness of the Incredible Years parenting program in The Netherlands. We capitalized on the large combined sample size, and on the extensive variability in family and child characteristics in these trials, to test whether five often hypothesized moderators actually impact the effects of Incredible Years on children’s conduct problems.

## Methods

### Procedure

Individual family level and item level data from all trials on the Incredible Years parenting intervention in The Netherlands were requested from the principal investigators. All investigators agreed to share their data. Table 1 provides an overview of the included trials. Three trials (#2, #3, and #4) were randomized controlled trials; one trial was a matched control group trial (#1). Two trials (#1 and #4) were indicated prevention; one trial (#2) was selective prevention; one trial (#3) was a mix of selective prevention and treatment. Control conditions were a no-treatment control condition (#1 and #2), a mix of no-treatment and care as usual (#4; around half of the families received alternative services) and a waiting list control condition (#3). Participants from all trials signed informed consent and study protocols were approved by Internal Review Boards (#1, #3 and #4 by a medical ethical committee; #2 by a Faculty of Social Sciences ethical committee).

**Table 1:** Overview of trial and family characteristics at baseline

	Trial #1 (Posthumus et al. 2012)	Trial #2 (Menting et al. 2014)	Trial #3 (Leijten et al. 2017)	Trial #4 (Weeland et al. 2017)
Trial characteristics				
Design	Matched control group	RCT	RCT	RCT
Number of families	144	99	156	387
IY version	Basic + advanced	Basic + home visits	Basic	Basic
Number of sessions offered; *M*	18	16	14.46^a^	14
Study focus	Indicated prevention	Selective prevention	Selective prevention & Treatment	Indicated prevention
Family demographics				
Child age; *M* (*SD*)	4.23 (2.87)	6.31 (2.69)	5.60 (1.34)	6.31 (1.33)
Child gender (% boys)	71%	52%	62%	55%
Educational level^b^; *M* (*SD*)	3.57 (.95)	1.78 (.94)	2.68 (1.16)	3.47 (1.03)
% ethnic minority	5%	78%	65%	11%
Problem severity				
ECBI conduct problems T1; *M* (*SD*)	129.88 (26.98)	110.09 (31.13)	124.17 (33.03)	133.27 (19.24)
ECBI conduct problems T2; *M (SD)*	122.30 (27.99)	98.95 (23.42)	114.57 (31.35)	125.69 (19.32)
Associated problems				
SDQ ADHD symptoms; *M* (*SD*)	4.59 (2.75)	4.42 (2.85)	5.71 (2.72)	5.79 (2.67)
SDQ emotional problems; *M* (*SD*)	2.15 (2.08)	2.52 (2.13)	3.12 (2.34)	3.32 (2.40)

*M* mean, *SD* standard deviation, *IY* Incredible Years, *RCT* randomized controlled trial, *ECBI* Eyberg Child Behavior Inventory, *SDQ* Strengths and Difficulties Questionnaire

^a^Trial #3 included multiple versions of the IY BASIC program because Incredible Years guidelines about program length changed during this trial

^b^Educational level categories were coded as 1 = primary education or less, 2 = secondary education, 3 = intermediate vocational, 4 = higher vocational, 5 = university

### Participants

The combined sample included 786 families (452 intervention; 334 control). Children ranged between 2 and 10 years (*M* = 5.79; *SD* = 1.66). Distribution of parental educational level approached normality (11% primary school or less, 20% secondary school, 28% intermediate vocational, 28% higher vocational, 13% university). Twenty-nine percent of the families had an ethnic minority background (see Table 2 for an overview). Conduct problem severity varied widely with scores on the Eyberg Child Behavior Inventory (ECBI) ranging from 44 (2.4 SD below the population mean) to 213 (3.8 SD above the population mean; possible range 36–252; see Burns and Patterson 2001, for norm scores on the ECBI). Half of the children showed ADHD symptoms (22% subclinical and 25% clinical). See the National Health Institute Survey (NHIS 2001) for norm scores on the Strengths and Difficulties Questionnaire (SDQ). Around a third of the children showed emotional problems (10% subclinical; 19% clinical).

**Table 2:** Characteristics of families with different cultural backgrounds

Cultural background	*n* (%^a^)	Child age (months)	% girls	Parental education^b^	Conduct problems	ADHD symptoms	Emotional problems
Dutch	535 (68.1)	68.73	39	3.36	131.46	5.62	3.00
North African	65 (8.3)	68.21	41	2.44	117.64	5.00	3.33
Caribbean	53 (6.7)	71.28	47	2.04	117.73	4.50	2.72
Latin American	34 (4.3)	76.44	53	1.94	117.01	4.74	2.52
Turkish	30 (3.8)	66.65	40	3.10	100.90	4.15	2.20
East-European	19 (2.4)	75.47	37	3.11	132.40	6.37	2.79
Sub-Sahara African	14 (1.8)	71.68	57	3.09	131.08	5.08	3.38
West-European/US/Canadian	11 (1.4)	68.62	55	4.09	132.40	5.18	3.00
Asian	10 (1.3)	84.88	40	3.67	128.60	4.60	3.60
Middle Eastern	8 (1.0)	73.25	50	3.83	132.66	4.50	4.83

^a^The cultural background of seven parents (0.9% of the sample) was unknown. Percentages therefore count up to 99.1% instead of 100%

^b^Educational level: 1 = primary education or less, 2 = secondary education, 3 = intermediate vocational, 4 = higher vocational, 5 = university

### Measures

#### Conduct Problems

Primary parents reported on children’s conduct problems using the Intensity Scale of the ECBI (Eyberg and Ross 1978). This well-established scale includes 36 items on a seven-point Likert scale (1 = *never* to 7 = *always*) to indicate the frequency of various problem behaviors (e.g., noncompliance, rule breaking). The ECBI has adequate psychometric properties, also in Dutch samples (Abrahamse et al. 2015). Internal consistency in our pooled sample was *α* = .90 at baseline and *α* = .91 at post intervention.

#### Parental Educational Level

Primary parents reported on their highest completed educational level. The same categories were used in all trials: primary education (coded as 1), secondary education (coded as 2), intermediate vocational (coded as 3), higher vocational (coded as 4), and university (coded as 5).

#### Parental Ethnic Background

Primary parents reported on their ethnic background. Trial #1 did not have data on parental ethnic background. For families from this trial, we therefore used information about the child’s ethnic background to estimate the primary parent’s ethnic background. Data were dichotomized into ethnic majority background (coded as 0) versus ethnic minority background (coded as 1).

#### ADHD Symptoms

The Hyperactivity and Inattention scale of the SDQ (Goodman 1997) was used to assess children’s baseline levels of ADHD symptoms. This scale includes five items on a three-point Likert scale (0 = *not true*, 1 = *somewhat true*, 2 = *certainly true*) and has good reliability and validity in Dutch samples (Van Widenfelt et al. 2003). Trial #1 did not include the SDQ but included the five items of the Inattention Problems scale of the Child Behavior Checklist (CBCL; Achenbach and Rescorla 2000). The Inattention Problems scale of the CBCL and the Hyperactivity and Inattention scale of the SDQ are known to correlate well (e.g., *r* = .71, Goodman and Scott 1999). We converted CBCL Inattention Problems scores into norm deviation scores using CBCL norm scores for preschool children (Achenbach and Rescorla 2000; i.e., values reflect the number of standard deviations the child scores above or below the population norm), which in turn were converted to SDQ Hyperactivity and Inattention scores using SDQ norm scores for children aged 2 to 7 (NHIS 2001). Internal consistency ranged *α* = .78 to *α* = .80 across trials and time points.

#### Emotional Problems

The Emotional Problems scale of the SDQ (Goodman 1997) was used to assess children’s baseline levels of emotional problems. This scale includes five items on a three-point Likert scale (0 = *not true*, 1 = *somewhat true*, 2 = *certainly true*). Trial #1 included the 36 items Internalizing scale of the CBCL (Achenbach and Rescorla 2000). The Internalizing scale of the CBCL and the Emotional Problems scale of the SDQ are known to correlate well (e.g., *r* = .74, Goodman and Scott 1999). We converted CBCL Internalizing scores into norm deviation scores using CBCL norm scores for preschool children (Achenbach and Rescorla 2000; i.e., values reflect the number of standard deviations the child scores above or below the population norm), which were then converted to SDQ Emotional Problems scores using SDQ norm scores for children aged 2 to 7 (NHIS 2001). Internal consistency ranged *α* = .64 to *α* = .67 across trials and time points.

### Intervention

Incredible Years (Webster-Stratton 2001) was used in its original form (i.e., not culturally adapted, except for translation of materials to Dutch). Parents participated in 12 to 18 weekly group sessions. Specifically, one trial offered the ADVANCED sessions (Webster-Stratton 2002) in addition to the BASIC sessions and one trial offered four additional home visits (see Table 1). Core components of the intervention include child-led play; use of praise and rewards to reinforce positive child behavior; effective limit setting; non-violent disciplining behavior (e.g., ignore and time-out); and coaching children’s social, emotional, and academic skills. Methods to teach parents these techniques include videotaped examples of parent-child interactions, brainstorms, and discussions about the pros and cons of different parenting techniques, and role-plays. Parents were given a book (Webster-Stratton 2006) and were encouraged to practice at home and to have weekly telephone contact with another parent from the group. At least one of the two group leaders of each group was a certified Incredible Years group leader. Program fidelity was monitored in each trial by videotaping the sessions and by using these videotaped sessions in frequent supervision meetings. Across trials, 16% of the families in the intervention condition did not attend any session. Families who did participate attended on average 68 to 79% sessions across trials. Data from all families were included in the analyses.

### Analytic Strategy

Multilevel analyses were performed in HLM 6.08 (Raudenbush et al. 2004) to account for the multilevel structure of the data. Specifically, families (level 1) were nested in Incredible Years groups (level 2). Children’s level of conduct problems (ECBI) immediately post intervention was the outcome variable in all models. For all models, full maximum likelihood estimation was used. Analyses controlled for trial level variance in intervention effectiveness and followed intention-to-treat principles (i.e., using data from all families, including from families that had not attended any session).

In all models, except for the intercept-only model, pretest ECBI scores (individual family level) and Trial (trial level) were included as control variables, in which Trial #4 served as reference group for Trial. Trial and the intervention variable (intervention [1] versus control [0]) were entered at the Incredible Years group level, whereas baseline conduct problem scores (ECBI), parental educational level, ethnic background (minority versus majority), ADHD symptoms (SDQ), and emotional problems (SDQ) were entered at the family level. To examine putative moderators of intervention effects, first the predictors of the family level were allowed to vary between groups by adding a variance component to the regression coefficient of the first level. When the regression coefficients for a particular family level predictor varied between Incredible Years groups, a cross-level interaction between this predictor and the intervention variable was added to the model.

All continuous variables were added grand mean-centered and all dichotomous variables dummy-coded to the model. Assumptions were checked, and the final model was run both with and without one outlier at the family level and two at the group level, providing similar results for all relevant outcomes.

## Results

### Preliminary Results

The overall effect of the intervention on children’s conduct problems was *d* = − 0.34 (95% CI − 0.49 to − 0.19), indicating lower levels of conduct problems in children of families in the intervention condition relative to the control condition. As expected, there was substantial variation in the extent to which families benefited from the intervention: reliable change indexes of families in the intervention condition ranged − 7.94 to 6.23. On average, ECBI scores of children in the intervention condition changed from *M* = 127.56 (*SD* = 26.97) to *M* = 116.61 (*SD* = 24.39). ECBI scores of children in the control condition changed from *M* = 129.33 (*SD* = 25.11) to *M* = 125.14 (*SD* = 25.63).

Following reliable change index guidelines (Jacobson and Truax 1991), 29% of the children in the intervention condition versus 13% of the children in the control condition improved reliably. Three percent of the children in the intervention condition worsened reliably, versus 5% of the children in the control condition. Children who got reliably worse had on average lower baseline levels of conduct problems (*M*_ECBI_ = 103.94) than children who either showed no reliable change (*M*_ECBI_ = 125.52) or who got reliably better (*M*_ECBI_ = 144.09). Following clinical significance guidelines (Jacobson et al. 1984), there was a reduction of 14% in the intervention condition of children who scored above the 90th percentile of conduct problems (24% at pretest, 10% at posttest), relative to a reduction of 6% in the control condition (23% at pretest, 17% at posttest).

Group leader characteristics (i.e., number of years of clinical work, number of previously provided Incredible Years groups), and parental satisfaction with the group leader (Parent Satisfaction Questionnaire; Webster-Stratton et al. 1989) were unrelated to parenting program effects on children’s conduct problems (βs = −.08 to .00; *p*s > .05).

### Parental Educational Level and Ethnic Background

**Fig. 1:**
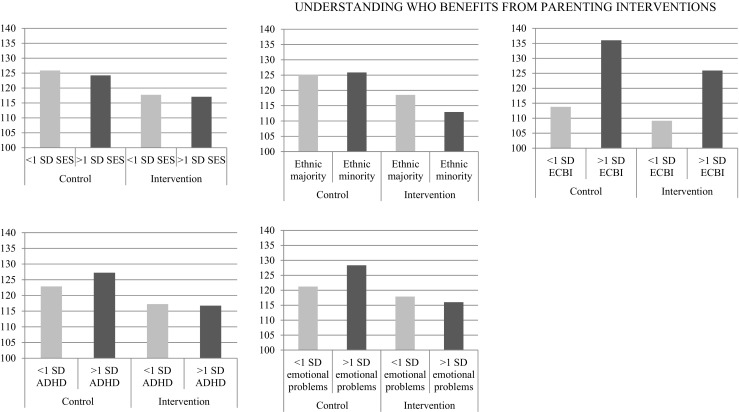
Socioeconomic status (i.e., educational level), Ethnic Minority Status, and Baseline Conduct Problems (i.e., ECBI), ADHD Symptoms and Emotional Problems as Moderators of Program Effects on Children’s Conduct Problems (Y-axis Reflects Post Intervention ECBI Scores Adjusted for Baseline ECBI scores)

The relation between parental educational level and post intervention conduct problems differed between Incredible Years groups (*σ*^2^ = 72.42, *χ*^2^ (27) = 56.02, *p* = .001), but these differences were not related to condition. Thus, parental education did not moderate the effects of Incredible Years on children’s conduct problems (*B* = − 1.35, *t*(46) = 0.66, *p* = .515; Fig. 1a).

Similarly, the relation between parental ethnic background and post intervention conduct problems differed between Incredible Years groups (*σ*^2^_=_ 6.66, *χ*^2^(47) = 78.34, *p* = .003), but these differences were not related to condition. Parental ethnic background did not moderate the effects of the parenting intervention on children’s conduct problems (*B* = − 6.68, *t*(46) = − 1.15, *p* = .257; Fig. 1b). Thus, there was no evidence to suggest that the intervention differently affected children with diverse educational or ethnic backgrounds.

### Conduct Problem Severity and Associated Problems

The relation between children’s baseline levels of problem severity and post intervention conduct problems differed between Incredible Years groups (*σ*^2^ = 0.02, *χ*^2^(46) = 70.92, *p* = .011), and differences were related to condition. Children’s baseline levels of conduct problem severity moderated intervention effects such that children with more severe conduct problems benefited more from the intervention in terms of reduced conduct problems (*B* = − 0.19, *t*(46) = − 2.31, *p* = .025; Fig. 1c).

The relation between children’s ADHD symptoms and post intervention conduct problems did not differ between Incredible Years groups (*σ*^2^ = 0.64, *χ*^2^(45) = 60.54, *p* = .061). Children’s ADHD symptoms did not moderate intervention effects (*B* = − 6.68, *t*(46) = − 1.15, *p* = .257). Children with higher levels of ADHD symptoms did not benefit less or more from the intervention than children with lower levels of ADHD (Fig. 1d).

The relation between children’s emotional problems and post intervention conduct problems differed between Incredible Years groups (*σ*^2^ = 2.04, *χ*^2^(46) = 73.76, *p* = .006) and differences were related to condition. Children’s emotional problems moderated intervention effects such that children with higher levels of emotional problems benefited more from the intervention in terms of reduced conduct problems (*B* = − 2.27, *t*(46) = − 2.56, *p* = .014). Specifically, children in the control condition with more emotional problems had higher levels of conduct problems than children in the control condition with fewer emotional problems, whereas children in the intervention conditions with either more or fewer emotional problems had similar levels of conduct problems (Fig. 1e).

### Sensitivity Analyses

Children’s conduct problems and emotional problems correlated *r* = 0.39. Entering both moderators in one model with robust standard errors did not change the study findings; entering both moderators in one model without robust standard errors slightly changed the study findings, such that only children’s emotional problems remained a significant moderator.

Eighteen per cent of the families in the intervention condition did not attend any intervention sessions. As Treated analyses on families who attended at least one of the sessions did not change any of the study findings based on Intention To Treat analyses.

In a post hoc sensitivity analysis, we used data from two out of the four trials to cross-check our moderator findings for immediate intervention effects against moderator findings for longer-term interventions effects. Trial #1 included a 12 month follow-up assessment (*n* = 137); Trial #4 included a 4 month follow-up assessment (*n* = 369). Longer-term data from Trial #2 were not available; longer-term data from Trial #3 could not be used because this trial used a wait list control design—by the time of their 3 month follow-up, families in the control condition had received the intervention. In the integrated data on longer-term effects from Trial #1 and Trial #4, we identified no additional moderators of intervention effects. Moreover, both moderators that were significant at immediate post-test (i.e., children’s baseline levels of problem severity and emotional problems) were no longer significant at follow-up. More detailed information on each of the sensitivity analyses is available upon request.

## Discussion

To strengthen prevention strategies, it is vital to identify families who benefit from parenting interventions, and families who may need alternative support. We used the extensive variability in family and child characteristics of 786 families from all trials on the Incredible Years parenting intervention in The Netherlands to test five of the most often hypothesized family and child characteristics as putative moderators of parenting intervention effects.

The overall effect size of Incredible Years on children’s conduct problems was *d* = − 0.34, similar to the effect size from a recent meta-analysis on Incredible Years (e.g., Menting et al. 2013). Families from a wide range of educational and ethnic backgrounds benefited equally. Levels of social and economic inequality in the Netherlands are generally low, which may have influenced our findings. That said, two of the four trials included families that face serious problems in terms of housing, employment, education, integration, and safety.

Children’s ADHD symptoms also did not moderate intervention effects. Children with co-occurring conduct problems and ADHD symptoms have worse prognoses than children with only conduct problems (Hinshaw et al. 1993; Rutter et al. 2006). Our finding that co-occurring ADHD symptoms did not diminish the effects of Incredible Years on children’s conduct problems is therefore potentially promising for prevention strategies. Two child characteristics did impact treatment success: children with more severe conduct problems and children with more emotional problems benefited more.

Children’s initial problem severity is the only moderator relatively consistently replicated across individual trials and meta-analyses in parenting intervention research (e.g., Leijten et al. 2013; Menting et al. 2013). Children with more severe problems have a larger scope for improvement and their parents may be more motivated to change. Children’s emotional problems as a moderator is less often studied. Our findings do not support the concern that children with co-occurring problems benefit less from interventions. In contrast, and similar to some other studies (e.g., Jarrett et al. 2014), our findings suggest that parenting interventions buffer the development of conduct problems particularly in children who suffer from co-occurring emotional problems.

If the main reason for not detecting moderator effects in smaller, individual trials is lack of statistical power, then why do we still find only two significant moderators, out of five often assumed moderators, in our well-powered combined sample of 786 families? To be sure, although several individual trials and meta-analyses find significant moderator effects, many more individual trials and meta-analyses do not find significant moderator effects (e.g., Ollendick et al. 2008; Reid et al. 2001; Weeland et al. 2017). Moreover, selective outcome reporting bias and publication bias may exist in that trials may have tested moderator effects, but did not report or publish their null findings (Dwan et al. 2008). Our well-powered analysis of a diverse sample, derived from multiple trials, suggests that of the relevant child and family characteristics tested, only severity of children’s behavior problems and children’s emotional problems moderate intervention success.

Because longer-term data were available for two out of four trials only, our sensitivity analyses on moderators of longer-term effects were less well-powered. Moreover, the two trials with available follow-up data differ meaningfully from the trials without available follow-up data: trials with available follow-up data mainly included white families with higher educational backgrounds, while the other two trials included an ethnically diverse sample of mothers released from incarceration (Trial #2), and an ethnically diverse sample with many socioeconomically disadvantaged families (Trial #3). We therefore interpret the findings of these post hoc sensitivity analyses with caution. That said, these sensitivity analyses confirm our main finding that often assumed moderators of parenting intervention effects are of limited help in explaining who benefits from parenting interventions for reducing disruptive child behavior. In addition, they suggest that while children’s baseline level of problem severity and emotional problems may help explain differential immediate treatment effects, they may not explain differential longer-term effects, or at least that their ability to do so may depend on the type of trials and families included.

The extent to which child and family characteristics such as educational level and ethnic minority background impact intervention effectiveness might in part depend on the specific intervention evaluated. Incredible Years is largely similar in content to other parenting interventions for children’s conduct problems (Kaehler et al. 2016). Different from most other interventions, however, Incredible Years uses a collaborative approach that might enhance cultural sensitivity. Group discussions, for examples, are used to encourage parents to come up with their own solutions and to use parenting techniques (e.g., play and praise) in a way that matches their personal values and norms (Webster-Stratton 2009). This inbuilt flexibility of the intervention may contribute to its ability to support families with different educational and ethnic backgrounds.

Yet, our study does confirm that intervention effects vary widely across families. If many of the often hypothesized moderators fail to explain this variation, then what makes some families benefit more than others? Perhaps moderators other than relatively “static” and seemingly general family characteristics (e.g., demographics) play a role. Theoretically more precise moderation research is needed to better illuminate differential intervention response trajectories. We highlight three possible directions for future research: first, parents’ individual interactions with the therapist might help explain how families’ intervention response trajectories develop (e.g., Kivlighan et al. 2014). Despite relatively strict Incredible Years therapist training and supervision procedures, families may experience different levels of alliance, trust, and/or support with therapists and other parents in the group. Thus, moderators may operate at the bidirectional therapist-client dyad level, rather than on the therapist or client level.

Second, families may respond differentially to different parenting techniques taught in an intervention, depending on, for example, the fit between specific techniques and parents’ values about these techniques and their previous experiences with these techniques. For example, parents may differ in how they feel about disciplining techniques such as time-out, and positive reinforcement techniques such as praise. Parenting interventions teach parents dozens of techniques (e.g., Leijten et al. 2015). Our abilities to understand families’ responses to parenting interventions might improve if we gain more insight into how families respond to the different parenting techniques taught in parenting interventions. This implies a shift in our research question from “who benefits?” to “who benefits from what?”

Third, family characteristics interact in predicting parenting intervention effects (e.g., Leijten et al. 2013). The traditional variable-centered approach to identify individual family characteristics that moderate intervention effects masks these interactions. Person-centered approaches that allow family characteristics to cluster in predicting response trajectories can help identify the families that benefit less or more from interventions (e.g., Pelham et al. 2017). Alternatively, recent trends to pool data across studies may allow for the statistical power needed to examine how family characteristics interact in predicting intervention effects.

Several limitations of our study merit attention. First, our analyses relied on parent-reported outcomes of child behavior. Parent reports can be biased because parents are not blind to experimental condition (Sonuga-Barke et al. 2013). As in any integrative data analysis study, our ability to include instruments depended upon inclusion of instruments in the individual trials. Teacher reported conduct problems and observed noncompliance were available only in a subset of trials and could therefore not be used as outcome measures in our integrative analysis. Please note that the primary aim of this study was to compare whether intervention effects varied for different subgroups of families, rather than to estimate the absolute magnitude of intervention effects for which this bias may have been of particular concern. Second, we included some of the most well-studied moderators but excluded several other potentially relevant child and family characteristics such as children’s executive functioning (e.g., Matthys et al. 2012) and biological markers of children’s differential susceptibility to environmental influences (e.g., Belsky et al. 2007). To our knowledge, however, none of these other putative moderators have (yet) shown a systematic, replicated pattern of differentiating between families who benefit more and families who benefit less. Third, three trials used the SDQ and one trial used the CBCL to assess children’s ADHD and emotional problems. We used established norms for both instruments to convert CBCL scores into SDQ scores, and subscales that are known to correlate well (Goodman and Scott 1999), but our approach does rely on the assumption that both instruments indeed measure the same construct.

We are among the first to integrate data across trials to allow for stringent moderation analysis of parenting intervention effects. Our findings suggest that at least in The Netherlands, the Incredible Years parenting intervention is not less or more effective for families with lower educational or ethnic minority backgrounds, or for children with ADHD symptoms. These rather different groups of families seem to benefit equally from the same intervention and these characteristics are unable to help differentiate between families who benefit more and families who benefit less. Our study does suggest that larger immediate intervention effects can be expected for children who show higher levels of conduct problems at the start of the intervention, and for children with more emotional problems. More generally, our study highlights that better understanding of moderators, and potentially the inclusion of different moderators, is needed to improve our understanding of who benefits from parenting interventions.
